# A Linear Relationship between Crystal Size and Fragment Binding Time Observed Crystallographically: Implications for Fragment Library Screening Using Acoustic Droplet Ejection

**DOI:** 10.1371/journal.pone.0101036

**Published:** 2014-07-02

**Authors:** Krystal Cole, Christian G. Roessler, Elizabeth A. Mulé, Emma J. Benson-Xu, Jeffrey D. Mullen, Benjamin A. Le, Alanna M. Tieman, Claire Birone, Maria Brown, Jesus Hernandez, Sherry Neff, Daniel Williams, Marc Allaire, Allen M. Orville, Robert M. Sweet, Alexei S. Soares

**Affiliations:** 1 Office of Educational Programs, Brookhaven National Laboratory, Upton, New York, United States of America; 2 Photon Sciences Directorate, Brookhaven National Laboratory, Upton, New York, United States of America; 3 Babylon Junior-Senior High School, Babylon, New York, United States of America; 4 Sayville High School, West Sayville, New York, United States of America; 5 Queens Metropolitan High School, Forest Hills, New York, United States of America; 6 Shoreham-Wading River High School, Shoreham, New York, United States of America; 7 Shelter Island High School, Shelter Island, New York, United States of America; 8 Purchase College, State University of New York, Purchase, New York, United States of America; 9 Freeport High School, Freeport, New York, United States of America; 10 Georgetown Day School, Washington, DC, United States of America; 11 Physics Department, University of Oregon, Eugene, Oregon, United States of America; 12 Department of Biomedical Engineering, Georgia Institute of Technology, Atlanta, Georgia, United States of America; 13 Department of Biological Sciences, University of Delaware, Newark, Delaware, United States of America; 14 Biosciences Department, Brookhaven National Laboratory, Upton, New York, United States of America; University of Oulu, Finland

## Abstract

High throughput screening technologies such as acoustic droplet ejection (ADE) greatly increase the rate at which X-ray diffraction data can be acquired from crystals. One promising high throughput screening application of ADE is to rapidly combine protein crystals with fragment libraries. In this approach, each fragment soaks into a protein crystal either directly on data collection media or on a moving conveyor belt which then delivers the crystals to the X-ray beam. By simultaneously handling multiple crystals combined with fragment specimens, these techniques relax the automounter duty-cycle bottleneck that currently prevents optimal exploitation of third generation synchrotrons. Two factors limit the speed and scope of projects that are suitable for fragment screening using techniques such as ADE. Firstly, in applications where the high throughput screening apparatus is located inside the X-ray station (such as the conveyor belt system described above), the speed of data acquisition is limited by the time required for each fragment to soak into its protein crystal. Secondly, in applications where crystals are combined with fragments directly on data acquisition media (including both of the ADE methods described above), the maximum time that fragments have to soak into crystals is limited by evaporative dehydration of the protein crystals during the fragment soak. Here we demonstrate that both of these problems can be minimized by using small crystals, because the soak time required for a fragment hit to attain high occupancy depends approximately linearly on crystal size.

## Introduction

Acoustic droplet ejection (ADE) [1] is an automated and keyboard-driven technology for growing protein crystals [2], improving the quality of protein crystals [3] and transferring protein crystals onto data collection media such as MiTeGen micro-meshes [4]. This method can also be used to discreetly position multiple two component systems onto one data collection micromesh or onto a moving conveyor belt that delivers each crystal into the X-ray beam [5]. Using this approach, a large fragment library can be rapidly screened for binding to protein crystals in a compact experiment. For example, by positioning 10 fragment containing crystals onto each micromesh, one conventional shipping Dewar would accommodate 1120 individual screening experiments [6]. Even greater specimen throughput is possible by separately transferring crystals and then fragments (which are contained in a large capacity source tray, such as a 1536 well plate) directly onto a moving conveyor belt system. The conveyor belt is mounted on a conventional goniometer for data collection, and it coordinates with a gated cryo stream system that flash freezes each specimen after the fragment has soaked into the crystal; consequently the maximum fragment screening speed is limited by the soaking time. Additionally, extended soak times may damage crystals by exposing them to the dehydrating effects of room air (unless the humidity is controlled, which is difficult to do for some high throughput methods).

Estimating the time required to soak each fragment into its crystal directly on the data collection media is a critical step in fast compact fragment library screening [7]. Previous research has identified important ligands that require extended soak times to reach a crystallographically observable occupancy [8]. For some of these proteins, it may not be possible to soak fragments directly on a micromesh and/or a moving conveyor belt (unless a method is devised to keep the crystals hydrated during the soak). Such cases can still be handled with acoustic high throughput screening methods, but the process will be slower and more laborious [9]. In other cases, theoretical calculations suggest that reducing crystal size may speed ligand binding [10]. Here, we use crystallographic methods to demonstrate that reducing the crystal size speeds fragment binding, thus enabling fast compact high throughput fragment screening (including using acoustic methods) in cases where the fragment soak times are otherwise prohibitively long.

## Methods

Undergraduate and high school research education programs in the Long Island area were selected as the primary personnel resource for carrying out this research. Interns learned to grow protein crystals, to measure crystal size, to soak crystals with ligands for a specified time, and to obtain diffraction data from the soaked crystals. Due to the number of specimens, a large research team was needed for specimen preparation, data collection, and data analysis. Interns generally worked in independent teams of two. Each team was “project aware and task competent,” meaning that they were aware of the overall goals of the entire group, but proficient in a specific set of tasks. A high value was placed on reproducibility, including control of pH and exact chemical composition of the mother liquor. We used monomeric N-acetyl glucosamine (NAG) binding to lysozyme and asparagine (ASN) binding to thermolysin as model systems. These systems were chosen due to their safety, relative ease of crystallization and long binding times. To prevent observer bias, all data analysis was performed in batch mode by a single command file that automatically processed through all 457 data sets in a systematic way.

### 2.1 Protein crystallization, X-ray diffraction data, and processing

X-ray diffraction data were obtained at NSLS beamlines X12C, X25, and X29 on lysozyme crystals soaked with 50 mM NAG and thermolysin crystals soaked with 100 mM ASN (crystallization conditions are displayed in Table 1). The soaking time was defined as the total time between when the crystals were combined with the ligand and when the crystals entered the liquid nitrogen (usually one intern prepared each specimen while the other intern measured the time). A full data set was collected from each crystal using ∼270 rotations of 1°. All data were obtained at 100 K and the X-ray exposure time was kept as low as possible to avoid radiation damage. The X-ray beam size was adjusted to match the crystal size. For moderate sized crystals (less than 120 µm) the X-ray beam size was adjusted by moving the slits. For larger crystals, the X-ray beam was defocused to make it larger. Data were processed with HKL2000 [11]. Data processing parameters are summarized in Table 2.

**Table 1:** Crystallization conditions.

	Lysozyme	Thermolysin
**Protein**	20–100 mg/ml	350 mg/ml
**Buffer**	20–100 mM NaAc pH 4.6	45% DMSO, 50 mM Tris pH 7.2–7.5, 1.4 M CaCl_2_
**Reservoir solution**	4%–8% NaCl, 10% Ethylene glycol	Water
**Cryo-protectant**	None	20% Ethylene glycol, 9% DMSO, 50 mM Tris pH 7.2–7.5, 0.28 M CaCl_2_
**Ligand Concentration**	50 mM	100 mM

**Table 2:** Crystallization, data collection, and model refinement statistics.

	Lysozyme	Thermolysin
**Ligand name**	N-acetyl glucosamine	Asparagine
**O_max_**	0.903	0.930
**K_d_^cryst^**	5.4 mM	7.5 mM
**τ (s/µm)**	0.794	0.284
**Mean |O_calc_−O_refine_|**	0.0976	0.0651
**R^2^ fit for O_calc_ to O_refine_**	77%	87%
**Crystal information**		
** Ligand concentration**	50 mM	100 mM
** Number of crystals**	354	103
** Space group**	P4_3_2_1_2	P6_1_22
** Solvent content**	38%	47%
**Unit-cell parameters (Å)**		
** a = b**	78.3±0.5	93.0±0.2
** c**	37.4±0.2	129.8±0.5
**Data collection and refinement parameters**		
** Resolution (Å)**	1.7±0.3	1.7±0.1
** Resolution I/σ_I_ = 5(Å)**	2.3±0.5	2.1±0.3
** Unique reflections**	15212	35994
** R_sym_(%)**	11.2%±4.6%	13.8%±4.3%
** Completeness (%)**	96.9%±5.1%	96.8%±6.4%
** R_work_ (%)**	18.2%±3.2%	14.8%±1.6%
** R_free_ (%)**	22.9%±4.2%	19.6%±2.2%
**R.m.s deviations from ideal**		
** Bond lengths (Å)**	0.016±0.011	0.014±0.005
** Bond angles (°)**	1.77±0.76	1.57±0.28
** No. of water molecules**	172±35	521±54
**Average B factor (Å^2^)**		
** Protein atoms**	18.6±5.9	16.6±5.4
** Water molecules**	31.0±4.5	33.8±4.4

X-ray diffraction data sets were obtained from 354 lysozyme crystals soaked with N-acetyl glucosamine and from 103 thermolysin crystals soaked with asparagine. Each X-ray data set was used to estimate the refined occupancy of the ligand (O_refine_) and a least squares procedure was used to fit Eq. 1 to these occupancies. The two fitted parameters were the occupancy at infinite time (O_max_, from which an intra-crystalline dissociation constant K_d_^cryst^ can be calculated using Eq. 2) and a fitting parameter related to diffusion speed (τ). For each measured value “x”, both the mean “x¯” and the population standard deviation “σ(x)” are listed as x¯ ± σ(x). The population standard deviation was calculated using the formula σ(x) = (Σ (x−x¯)^ 2^/n)^ ½^, where “n” is the number of measurements (always equal to 354 for lysozyme data and 103 for thermolysin data).

**Figure 1:**
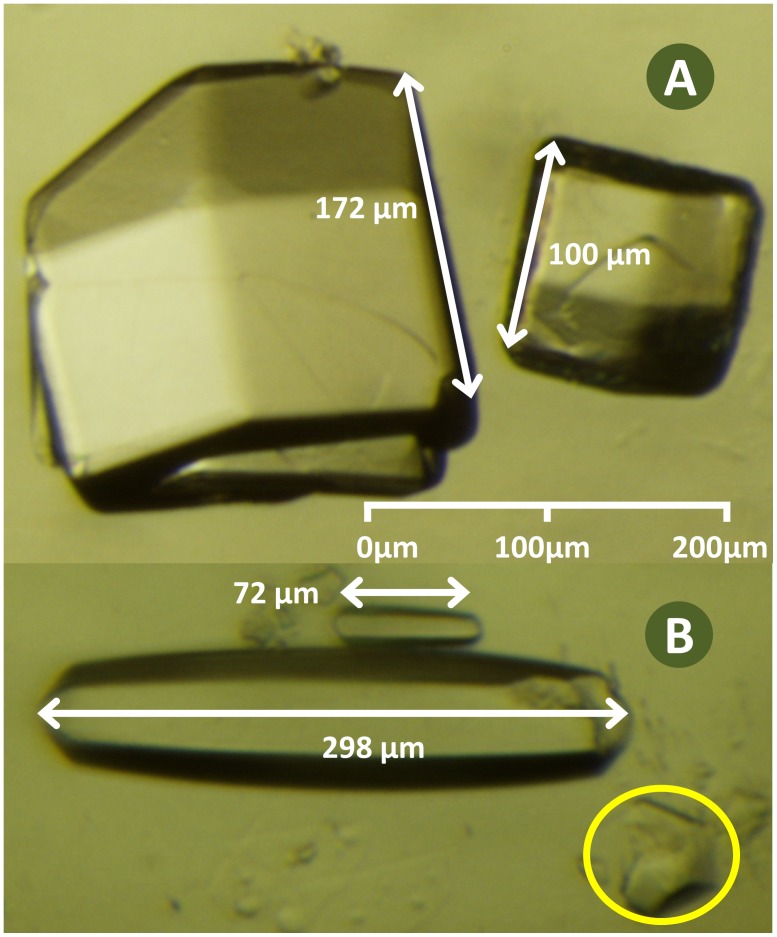
Lysozyme crystals have a cubic habit and thermolysin crystals have an elongated habit. Lysozyme forms cubic crystals which were measured along the longest sides as shown (panel A). One large and one medium sized lysozyme crystal are shown. Thermolysin crystals have an elongated habit and were measured along the long axis (panel B). One large and one small crystal are shown. Occasionally a small piece of a crystal broke off (yellow highlight). In these cases, the longest crystal fragment was measured (without adjusting the length to account for the missing piece). The soaking time should correlate with the shortest crystal dimension, but the short side is difficult to measure accurately. Fortuitously, it was possible to grow lysozyme and thermolysin crystals with a very consistent crystal habit. The long crystal axis (which was easy to measure) was a good proxy way to compare the short crystal axis (which was difficult to measure).

Because the project was carried out by many researchers over two years, a systematic approach to measuring crystal size was necessary. Crystal size was defined as the largest distance between parallel crystal faces (measured using the CellSans software on a Leica MZ16 microscope fitted with an Olympus DP72 camera). Chemicals would likely diffuse into the crystal most quickly along the smallest crystal dimension, but many of the crystals were too small for the smallest side to be visible. Crystals tended to settle under gravity so that they rested on their largest side, occluding the smallest side. Additionally, the smallest dimension is often poorly defined (for example, the width of the thermolysin crystals changes along its length because the crystal width is tapered along the long axis). In contrast, the largest side was usually well defined and was easier to measure accurately. The shape of lysozyme and thermolysin crystals was largely independent of size, so that the observations made using the (measured) long side are accurate for the (unmeasured) short side also. We reduced the ambiguity caused by variations in lysozyme crystal habit by selecting crystallization conditions that yielded uniform crystals with a cubic habit. Thermolysin crystals have a columnar habit and each crystal was measured along its long axis (Figure 1).

#### Lysozyme

Lysozyme from chicken egg white (Sigma-Aldrich L4919) was used to grow crystals at 22°C using hanging drop, micro-seeding, and micro-batch as needed to yield each desired crystal size (Table 1). This was fine-tuned by perturbing drop size, concentration of salt (4%–10% w/v) as well as the concentration of protein (20–100 mg/ml). The pH was maintained at 4.6 and buffered with sodium acetate (20–100 mM). Protein powder was dissolved in the buffer and then filtered through a 0.22 µm syringe filter and centrifuged at 1216 g for 10 minutes. Equal volumes of the protein and precipitant solutions were used in all hanging drop preparations. Microcrystals (under 30 µm) were obtained by using the batch method, gently agitated overnight in an orbital rocking platform. Micro seeding 0.1–1 µl of batch crystal solution into a 4 µl hanging drop (cover slip rinsed in 20% ethanol + water rinse) yielded 30–90 µm crystals. Larger crystals were grown by unseeded hanging drops. In total, 354 X-ray diffraction data sets were obtained from lysozyme crystals of various sizes treated with NAG for different amounts of time.

Lysozyme crystals were measured using the CellSans software, then picked up using a cryo-loop (Hampton HR4-955) and transferred onto a cover-slip containing a 1 µL drop of NAG-containing mother liquor (50 mM NAG) where they soaked for 0–900 seconds. The crystals were then picked up in the cryo-loop again and were flash cooled in liquid nitrogen. Glycerol is an effective cryo-protectant for lysozyme, but it was not used in order to prevent conflation of the data by competitive exclusion or concentration driving. Lysozyme contains six binding sites designated A–F [12]. NAG preferentially binds to site C, and glycerol binds to site D [13], but it is possible that a high concentration of glycerol may disrupt the ability of NAG to bind. To avoid the possibility of competitive exclusion, no cryo-protectant was used. Ice ring problems were controlled with careful mother liquor minimization (the crystallization solution is moderately cryo-protective). Minimization methods included carefully matching cryo-loops to crystal size and the natural improvement in technique that comes from practice.

#### Thermolysin

Thermolysin crystals were grown at 22°C using the hanging drop method, which produced crystals in the 200 µm range (Table 1). The micro-batch technique was used to reduce crystal size to as small as 50 µm. The protein solution consisted of 45% by volume DMSO, 50 mM Tris, 1.4 M CaCl_2_ and 350 mg/ml thermolysin. The pH of both the thermolysin and ASN solutions was kept within 7.2–7.5 by the Tris-buffered addition of HCl. For the standard crystallizing procedure, a 1 µl drop of protein solution was pipetted onto a glass slide and suspended over a reservoir of H_2_O. To produce smaller crystals, up to 3 µl of H_2_O were added to the original drop prior to sealing the slide onto the reservoir well. A total of 103 X-ray diffraction data sets were obtained from crystals ranging from 50–280 µm in length and 0–10 minutes in soaking time.

Thermolysin crystals were transferred into a ligand solution consisting of 100 mM ASN, 9% by volume DMSO, 50 mM Tris, 0.28 M CaCl_2_ and 20% by volume ethylene glycol (cryo-protectant). Crystals were then mounted onto loops or micro-meshes for data collection (micro-meshes were preferable for smaller crystals). Micromesh mounted crystals had mother liquor minimized by gently dabbing each mesh against a cloth soaked in ASN-containing mother liquor; this removed excess mother liquor while maintaining a moist environment for the crystals.

### 2.2 Occupancy calculation

The starting model for occupancy refinement was generated from water-stripped [1LYZ] [14] and [4M65] [6], refined by *REFMAC*, and hydrated with *ARP/wARP* solvent [15]. The starting model contained NAG with occupancy = 0.50 and B = 28 Å^2^ or ASN with occupancy = 0.50 and B = 34 Å^2^ (the target 28/34 Å^2^ temperature factors were averaged from models refined against data where full occupancy was achieved by overnight soak in 200 mM ligand). Unrecorded reflections were copied from the starting model with the NAG removed (electron counting methods based on the molecular envelope are sensitive to missing reflections) [16]. For each X-ray diffraction data set, the 1LYZ structure was stripped of waters, refined with *REFMAC*, hydrated with ARP/wARP solvent, and the NAG was removed. The CCP4 suite program *SFALL* was used to generate calculated structure factors and these were substituted for any missing reflections in the data (up to the 1.6Å/1.9Å diffraction limit used for all lysozyme/thermolysin crystals). On average, the reflections that were added in this way corresponded to 3.1% of all lysozyme crystal reflections and 3.2% of all thermolysin crystal reflections. For each of the 354 lysozyme + NAG data sets, and each of the 103 thermolysin + ASN data sets, a common procedure was used to calculate the occupancy of the ligand using the X-ray diffraction data in three different ways.

#### Three refined models with occupancy estimate

An atomic model with a refined occupancy for the N-acetyl glucosamine soaked into lysozyme crystals and for the asparagine soaked into thermolysin crystals was generated using *PHENIX* [17] and *REFMAC* [18]. For each data set, three methods were used to generate an atomic model with a refined ligand occupancy using the X-ray diffraction data. The first refined model was generated using *PHENIX* to simultaneously refine both the occupancy and the temperature factor of each ligand using all of the recorded X-ray diffraction data. A second refined model was generated using *PHENIX* to refine the occupancy of each ligand with the ligand atoms having a fixed atomic temperature factor (B_fixed_ = 28 Å^2^ for NAG atoms; B_fixed_ = 34 Å^2^ for ASN atoms). To decouple the occupancy of each atom from its temperature factor, each data set was modified so that all of the data sets had a common resolution limit (the resolution limit was 1.6 Å for lysozyme data and 1.9 Å for thermolysin data; data sets were truncated after applying a reciprocal space temperature factor such that the last shell I/σI was reduced to unity). A third refined model was generated using *REFMAC* to iteratively adjust the ligand atom occupancy until their atomic temperature factors refined to 28 Å^2^ (NAG atoms) or 34 Å^2^ (ASN atoms), using the same modified data described previously (the target temperature factors were determined by averaging the values observed in lysozyme crystals with full occupancy NAG and thermolysin crystals with full occupancy ASN).

#### Occupancy calculation

The best estimate for the occupancy of each ligand was made by integrating each ligand’s electrons from the electron density map. In the case of NAG, the electrons in the acetyl moiety were disregarded to prevent mis-calculation because of counting acetone electrons that can occupy the same site. In the case of ASN, electrons in the vicinity of the active site zinc atom were masked out of the integration region to prevent distortion by the very large electron density of the heavy atom. Each of the three coordinate files (with refined occupancies) was used to establish a mask containing the NAG region (minus the acetyl moiety) or the ASN region (with the zinc atom masked out). Three electron density maps were generated from the X-ray diffraction data (with phases from each of the three refined coordinate files described above). Each of the three electron density maps was placed on an absolute level by adjusting the F_000_ such that the observed count of electrons in the well-ordered protein envelope was equal to the known number of electrons in the protein. The total number of electrons in the volume occupied by each ligand was then integrated from each of the three electron density maps. For each of the three refined atomic coordinate files, the occupancy of the ligand was estimated by dividing the observed integrated electron count by the known number of electrons in the ligand [19] (both the observed electrons in the ligand envelope and the known electrons in the ligand were adjusted by subtracting the contribution from ordered waters that bind in the absence of ligand). This procedure is described in unpublished data (Soares & Casper et al). Briefly, known information about the topography surrounding the ligand can be accounted for in real space, but not in reciprocal space. For example, the asparagine ligand binds near to a zinc atom in thermolysin. Reciprocal space refinement of the asparagine occupancy will occasionally be confounded by spill over from the zinc atom. In real space the zinc atom contribution can be flattened prior to electron counting. A similar accounting problem occurs when N-acetyl glucosamine binds to lysozyme by displacing an acetate ion. Accounting for these contributions requires an accurate model of the vicinity of a well described binding site, so electron counting methods would not be appropriate for ligand discovery purposes.

A final best occupancy was obtained for each data set by averaging the three occupancy numbers obtained by the electron integration procedure phased with the three refined atomic models. The largest observed standard deviation between these three estimates was 22.41% for lysozyme and 22.36% for thermolysin. The median standard deviation between the three estimates was 6.51% for lysozyme and 7.36% for thermolysin. The average standard deviation between the three estimates was 6.93% for lysozyme and 7.63% for thermolysin. The minimum allowed occupancy was 0.01, and the maximum was 0.99.

To determine the precision and accuracy of our electron counting techniques, we obtained X-ray diffraction data from twenty similarly sized crystals (∼100 µm), half of which were ASN-free controls and half of which were soaked overnight in 100 mM ASN.

## Results

**Figure 2:**
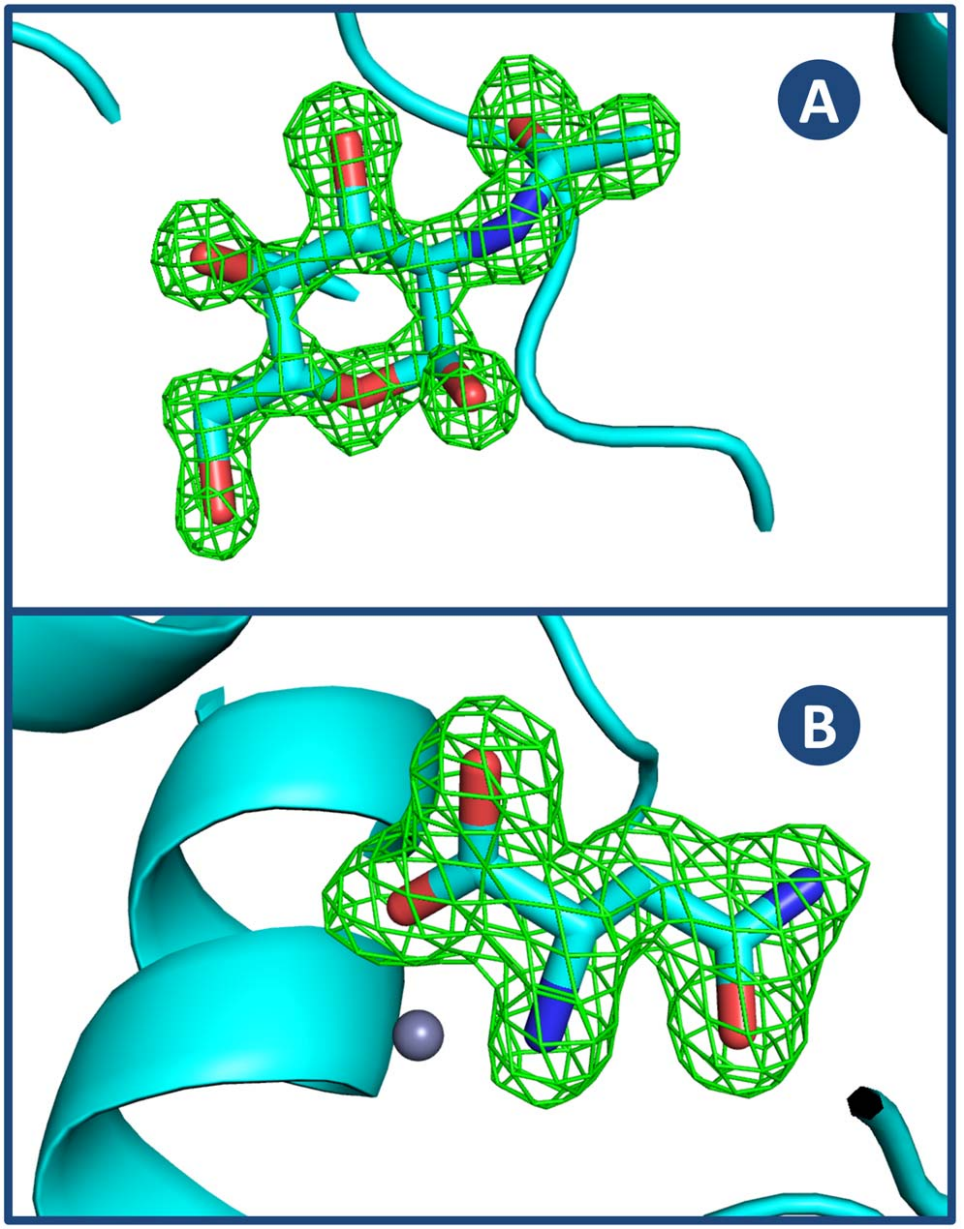
Electron density for NAG bound to lysozyme and for ASN bound to thermolysin. Panel A: N-acetyl glucosamine is shown bound to lysozyme (difference omit map is contoured at 3.0 σ). The lysozyme data comes from a 310 µm crystal that was soaked for 750 seconds, with a refined occupancy of 74% and occupancy calculated using Eq. 1 of 68%. Panel B: Asparagine is shown bound to thermolysin (difference omit map is countered at 3.0 σ). The thermolysin data comes from a 220 µm crystal that was soaked for 601 seconds, with a refined occupancy of 99% and occupancy calculated using Eq. 1 of 84%.

**Figure 3:**
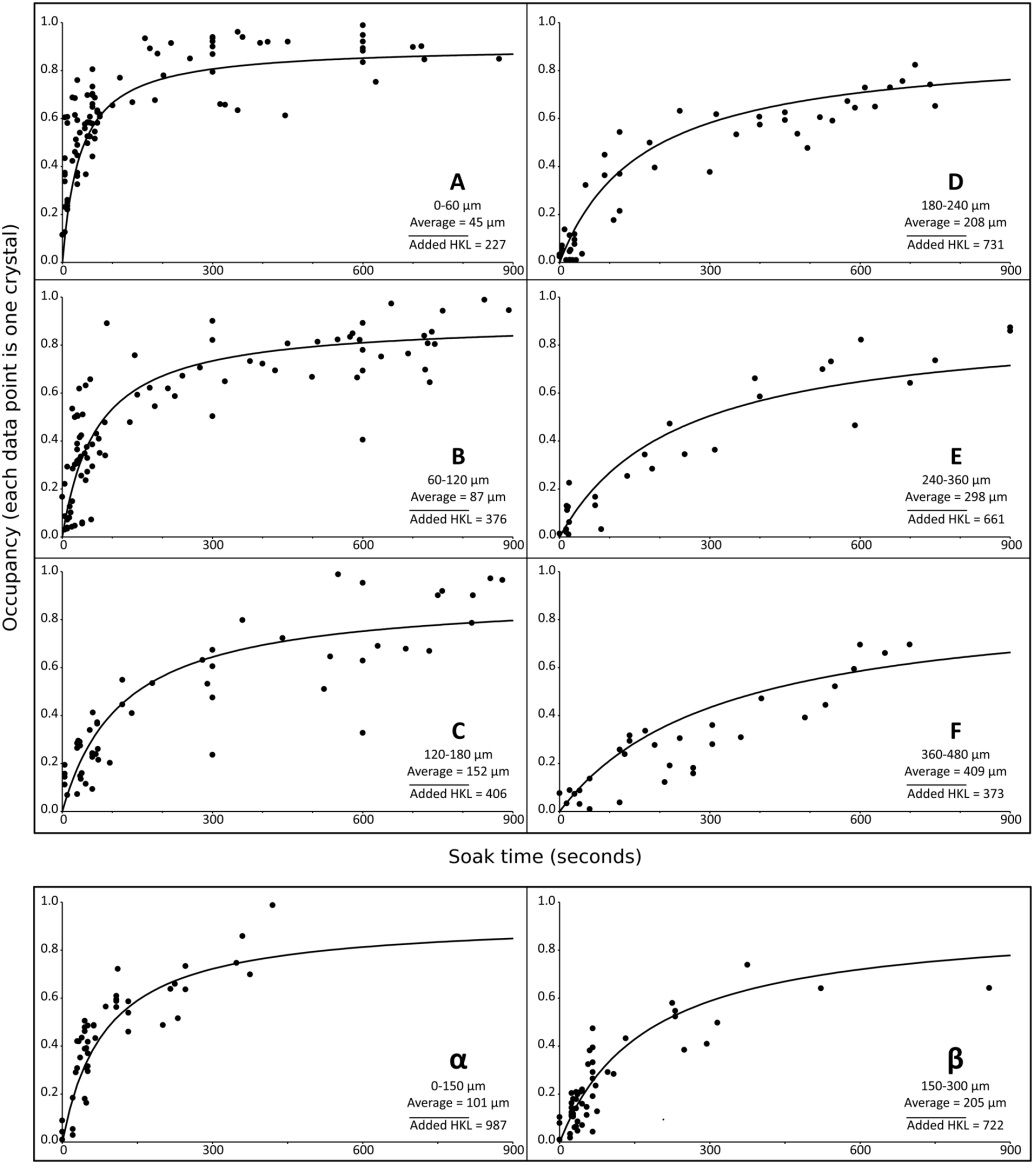
Refined occupancies (y axes, %) as a function of soak time (x axes, seconds). Two dimensional slices are shown for the three dimensional relationship between crystal size, ligand soak time, and occupancy (O_calc_ and O_refine_). In each panel, the crystal size variable is excluded by grouping crystals of similar sizes. Lysozyme + NAG crystals are grouped by size (0–60 µm in box **A**, 60–120 µm in box **B**, 120–180 µm in box **C**, 180–240 µm in box **D**, 240–360 µm in box **E**, 360–480 µm in box **F**). Thermolysin + asparagine crystals are grouped into two sizes (0–150 µm in box **α**, and 150–300 µm in box **β**). Each data point represents the observed soak time and occupancy of one crystal + ligand. The average size for crystals in each range is indicated. The average number of calculated structure factors that were added into the data (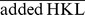) is also shown (larger crystals had more overloads and consequently more added reflections). Inspection of the relationship between soak time and refined occupancy revealed a linear relationship between crystal length and the time needed to reach 50% maximum occupancy (t_1/2_), so that t_1/2_ = Lτ, where L is the crystal length and τ is a fixed constant. Best fits for lysozyme (R^2^ = 78%) and thermolysin (R^2^ = 88%) were calculated using least squares applied to Eq. 1. In each panel, a solid line shows Eq. 1 with the average size of crystals in that panel assigned to L (fitting parameters taken from Table 2). Note that the data in each panel come from crystals with similar but not identical sizes. Consequently, the data fit Eq. 1 much better than these graphs suggest. The average residual between calculated occupancies from Eq. 1 and refined occupancies from the X-ray diffraction data was 9.76% for lysozyme + NAG and 6.51% for thermolysin + ASN.

A total of 354 lysozyme + NAG data sets and 103 thermolysin + ASN data sets were obtained from crystals of various sizes soaked with their ligands for different times. The orientations for NAG binding to lysozyme and ASN binding to thermolysin are shown in Figure 2 (for clarity, these were among the best of our crystals). For both lysozyme + NAG and thermolysin + ASN, inspection of the relationship between soak time and observed occupancy revealed a linear relationship between the time needed to reach 50% maximum occupancy and the size of the crystal. The experimental occupancy was observed to fit the following asymptotic curve (Figure 3):
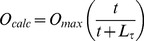
(1)


Where L is the crystal length, t is the soak time, and the calculated occupancy O_calc_ is fit to the experimentally refined occupancy O_refine_. The two fitting parameters were O_max_ (the limit occupancy after a long soak) and τ (a fitting parameter with unit s/µm). A least squares algorithm was used to calculate the values of O_max_ and τ which resulted in the best overall fits to the data sets for lysozyme (O_max_ = 90%, τ = 0.79 s/µm) and for thermolysin (O_max_ = 93%, τ = 0.28 s/µm). The average absolute differences between the refined and calculated occupancy values were also calculated (9.76% for lysozyme and 6.51% for thermolysin). A plot of O_refine_ versus O_calc_ fits well to a straight line with unity slope (R^2^ = 77% for lysozyme and 87% for thermolysin). This indicates that Eq. 1 explains 77% of the variance in O_refine_ for lysozyme and 87% for thermolysin. The results are summarized in table 2.

An intra-crystalline dissociation constant K_d_^cryst^ is also reported in table 2. The K_d_^cryst^ was calculated from the refined O_max_ using the fraction saturation equation [20]:
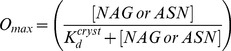
(2)


In the case of lysozyme binding to N-acetyl glucosamine, the calculated K_d_^cryst^ [21] dissociation constant is similar to values reported for free ligand [22]. The dissociation constant for thermolysin binding to asparagine has not been reported, and we estimate it to be 7.5 mM using the O_max_ obtained from Eq. 1. However, there may be significant uncertainty in this estimate (Table 3).

**Table 3:** Precision and accuracy of the occupancy calculation and the K_d_^cryst^ value.

	Refined Occupancy (%)		Deduced
	0 mM ASN	100 mM ASN	K_d_^cryst^ (mM)
**Phenix Occ. + B**	63±8	90±6	11
**Phenix Occ. only**	60±8	98±2	2
**Avg. e^−^ count (3 models)**	3±2	85±9	17
** Model 1: Phenix Occ. & B**	4±3	88±9	13
** Model 2: Phenix Occ. only**	5±5	86±8	16
** Model 3: Refmac**	1±1	82±11	22

X-ray diffraction data were obtained from 10 thermolysin crystals that were not soaked in asparagine (first column) and from 10 thermolysin crystals that were soaked in 100 mM asparagine overnight (second column). We disregard crystal size because of the long soak times (all crystals were approximately 100 µm). For each group of ten crystals, the average and standard deviation for the refined occupancy are shown separately for each of the methods used for the refinement (two conventional *PHENIX* refinements, three electron counting methods described in §2.2, and the average of these three). Since the crystals were soaked overnight (t→∞ so that O_max_ = O_refine_) the intra-crystalline dissociation constant K_d_^cryst^ is readily obtained from O_max_ using Eq. 2 (shown in the third column) [20]. There is a significant discrepancy between the K_d_^cryst^ value obtained from the curve fitted O_max_ (7.5 mM) and the value from the overnight soak O_max_ (17 mM). The occupancy refinement protocols all have higher precision (as seen by the low standard deviation) than accuracy. This high precision is sufficient to demonstrate that smaller crystals reach high occupancy faster. We report K_d_^cryst^ to confirm that the binding affinity is within the expected range for a small molecule product, but with significant uncertainty. We did not perform a similar analysis for lysozyme binding to N-acetyl glucosamine because the value obtained from the curve fitted O_max_ (5.4 mM) was very close to reported values (4–6 mM) [22].

**Figure 4:**
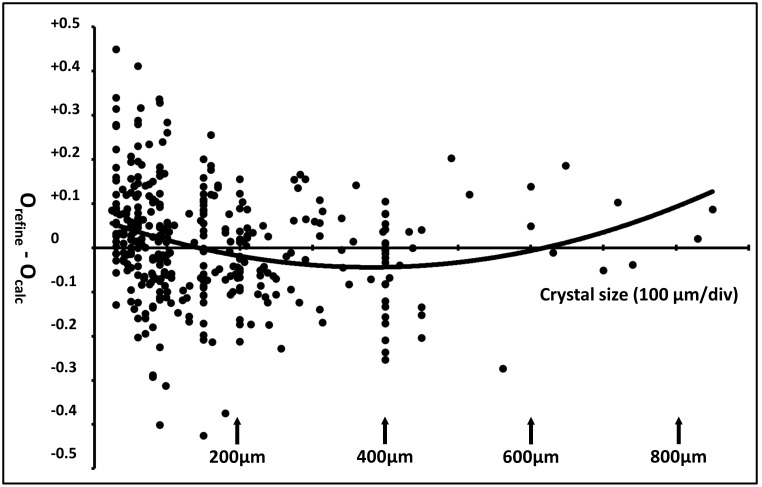
Higher order terms not accounted for by Eq. 1 may exist. The residual (O_refine_−O_calc_) between refined occupancies and calculated occupancies as a function of crystal size (µm) for lysozyme + NAG suggests that small crystals may bind even faster than predicted by Eq. 1. Each crystal data set is represented as one point (x-axis hash marks represent 100 µm of crystal size). The average absolute difference between refined occupancy and calculated occupancy is 9.73%. A polynomial best fit to the residual (solid line) indicates that there may be higher order terms (R^2^ = 6%). If Eq. 1 fully described the relationship between ligand occupancy, soaking time, and crystal size then the residual should show shapeless noise. Ten very large crystals (over 480 µm) were soaked with NAG to further investigate the size dependence of the discrepancy (these data points are on the right side of the figure, and were not used for any other purpose). This possible limitation of Eq. 1 finds weak support in the data; we do not assert that it is the best or only evidence that Eq. 1 is incomplete. Despite these possible limitations, we believe that Eq. 1 relates soak time and crystal length sufficiently to help plan high throughput screening experiments.

Eq. 1 is adequate for choosing an appropriate crystal size for each soaking experiment, but there may be contributions such as electrostatic steering, cooperativity, ligand affinity, conformational changes, or complex ligand interactions that are not accounted for. If Eq. 1 accounted for all of these factors, then the discrepancy between the occupancy calculated from Eq. 1 (O_calc_) and the occupancy refined from the data (O_refine_) would be shapeless noise. However, plotting the discrepancy O_refine_−O_calc_ as a function of crystal size (Figure 4) shows small systematic differences (superposed on larger randomly distributed differences). Crystals smaller than 200 µm appear to bind even more rapidly than predicted using Eq. 1. If real, this systematic error may be experimental (for example, interns may over-estimate the size of the smallest crystals) or it may be physical (for example, ligand depletion may lower the refined occupancy of large crystals). We acknowledge that Eq. 1 is only an approximation. A more accurate prediction of binding time could be obtained using a higher order polynomial expansion in Eq. 1.

## Discussion

We used lysozyme binding to NAG and thermolysin binding to ASN as model systems to investigate the correlation between soak time, crystal size, and crystallographically refined occupancy. Our results demonstrate that smaller crystals can be used to decrease the time needed for fragments to soak into the crystals. In some cases smaller crystals may be used so that binding speed can keep up with fast specimen preparation techniques such as acoustic droplet ejection. Other applications include accelerating very slow ligand-exchange protocols [8].

Our models suggest that for any desired occupancy, there is a direct proportionality between crystal size and the soak time required for the NAG or ASN ligand to reach that occupancy. These models (Eq. 1, Table 2) agree with experimental data to within a mean absolute deviation of 9.76% and 6.51% for lysozyme + NAG and thermolysin + ASN, respectively. Lysozyme crystals pack tightly and form small protein channels that restrict fragment mobility (∼1 nm wide) [23]. NAG binds to lysozyme with moderate affinity which will increase the time until observable binding (K_d_ = 4–6 mM) [22]. The narrow channels in lysozyme and the modest binding of NAG make this a conservative model system to examine cases where on-micromesh or on-conveyor binding studies are likely to result in crystallographically detectable occupancy. In contrast, the thermolysin crystal lattice contains fewer constrictions.

Fragment libraries can be screened by using acoustic droplet ejection to combine crystals and fragments directly on micro-meshes or on a moving conveyor belt. On micro-meshes, as many as 10 protein crystals can be combined with 10 different fragments [6]. An unlimited number of crystal + fragment screens can be combined on a conveyor belt [5]. These techniques efficiently use fragment chemicals (∼2.5 nL per screened condition), protein (∼25 nL per screened condition), space (1120 screened conditions per standard shipping Dewar; no limits using a conveyor belt), and synchrotron beam time (<1 second/screened condition) [24]. Evaporative dehydration of the protein crystal limits these fragment screening applications to systems where the fragment soak time is not prohibitive. Slow-binding compounds can be screened (without time constraint) in trays using ADE, but will consume significantly more resources such as purified protein and chemical compounds (∼1 µl per screened condition). Hence, it is desirable to identify promising cases where the cost-efficient on-micromesh or on-conveyor soaking methods are adequate.

If the lysozyme and thermolysin results are generally applicable such that the time needed for chemicals to soak into many protein crystals is correlated with the crystal size, it should be possible to use small crystals to reduce the problem of crystal dehydration during prolonged soaking and also to accelerate high throughput screening projects. For some high throughput screening strategies, fragments are soaked directly on the data collection media prior to cryo-plunging. Crystallographically observable occupancy must be achieved before evaporative dehydration. If the soak time is too long, desiccation will destroy the crystalline order before the fragment is detectable. This is especially relevant to crystals that exhibit extended soak times (due to tight packing or other reasons such as having one or more narrow constrictions). If crystals are soaked with chemicals *in situ* on a direct injection system such as a crystal conveyor belt, reducing the time needed for each chemical to reach high occupancy in the crystal lattice will directly enhance the screening speed.

## References

[1] Ellson R, Mutz M, Browning B, Lee L, Miller MF, et al (2003) Transfer of low nanoliter volumes between microplates using focused acoustics–automation considerations. Journal of the Association for Laboratory Automation 8(5): 29–34.

[2] Villasenor AG, Wong A, Shao A, Garg A, Donohue TJ, et al (2012) Nanolitre-scale crystallization using acoustic liquid-transfer technology. Acta Crystallographica D 68: 893–900. 10.1107/S0907444912016617 PMC3413209 22868754

[3] Villasenor AG, Wong A, Shao A, Garg A, Kuglstatter A, et al (2010) Acoustic matrix microseeding: improving protein crystal growth with minimal chemical bias. Acta Crystallographica D 66: 568–576. 10.1107/S0907444910005512 20445232

[4] Soares AS, Engel MA, Stearns R, Datwani S, Olechno J, et al (2011) Acoustically mounted microcrystals yield high-resolution X-ray structures. Biochemistry 50: 4399–4401. 21542590 10.1021/bi200549x PMC3144476

[5] Roessler CG, Kuczewski A, Stearns R, Ellson R, Olechno J, et al (2013) Acoustic methods for high-throughput protein crystal mounting at next-generation macromolecular crystallographic beamlines. Journal of Synchrotron Radiation 20(5): 805–808. 23955046 10.1107/S0909049513020372 PMC3747951

[6] Yin X, Scalia A, Leroy L, Cuttitta CM, Polizzo GM, et al (2014) Hitting the target: fragment screening with acoustic in situ co-crystallization of proteins plus fragment libraries on pin-mounted data collection micromeshes. Acta Crystallographica D 70(5): 1177–1189. 10.1107/S1399004713034603 PMC4014116 24816088

[7] Erlanson DA, McDowell RS, O'Brien T (2004) Fragment-based drug discovery. Journal of Medicinal Chemistry 47(14): 3463–3482. 15214773 10.1021/jm040031v

[8] Collins PM, Hidari KI, Blanchard H (2007) Slow diffusion of lactose out of galectin-3 crystals monitored by X-ray crystallography: possible implications for ligand-exchange protocols. Acta Crystallographica D 63(3): 415–419. 10.1107/S090744490605270X 17327679

[9] Cipriani F, Rower M, Landret C, Zander U, Felisaz F, et al (2012) CrystalDirect: a new method for automated crystal harvesting based on laser-induced photoablation of thin films. Acta Crystallographica D 68: 1393–1399. 10.1107/S0907444912031459 22993093

[10] Geremia S, Campagnolo M, Demitri N, Johnson LN (2006) Simulation of diffusion time of small molecules in protein crystals. Structure 14(3): 393–400. 16531224 10.1016/j.str.2005.12.007

[11] Otwinowski Z, Minor W (2001) Denzo and Scalepack. Crystallography of Biological Macromolecules, 2.

[12] Rand-Meir T, Dahlquist FW, Raftery MA (1969) Use of synthetic substrates to study binding and catalysis by lysozyme. Biochemistry 8(10): 4206–4214. 5346398 10.1021/bi00838a044

[13] Tanley SW, Schreurs AM, Kroon-Batenburg LM, Meredith J, Prendergast R, et al (2012) Structural studies of the effect that dimethyl sulfoxide (DMSO) has on cisplatin and carboplatin binding to histidine in a protein. Acta Crystallographica D 68(5): 601–612. 10.1107/S0907444912006907 22525758

[14] Diamond R (1974) Real-space refinement of the structure of hen egg-white lysozyme. Journal of molecular biology 82(3): 371–391. 4856347 10.1016/0022-2836(74)90598-1

[15] Langer G, Cohen SX, Lamzin VS, Perrakis A (2008) Automated macromolecular model building for X-ray crystallography using ARP/wARP version 7. Nature Protocols 3(7): 1171–1179. 18600222 10.1038/nprot.2008.91 PMC2582149

[16] Soares AS, Caspar DL, Weckert E, Heroux A, Holzer K, et al (2003) Three-beam interference is a sensitive measure of the efficacy of macromolecular refinement techniques. Acta Crystallographica D 59(10): 1716–1724. 10.1107/s0907444903015403 14501109

[17] Adams PD, Afonine PV, Bunkoczi G, Chen VB, Davis IW, et al (2010) PHENIX: a comprehensive Python-based system for macromolecular structure solution. Acta Crystallographica D 66(2): 213–221. 10.1107/S0907444909052925 PMC2815670 20124702

[18] Winn MD, Murshudov GN, Papiz MZ (2003) Macromolecular TLS refinement in REFMAC at moderate resolutions. In: Methods in Enzymology 374 pp. 300–321. 10.1016/S0076-6879(03)74014-2 14696379

[19] Yu B, Blaber M, Gronenborn AM, Clore GM, Caspar DLD (1999) Disordered water within a hydrophobic protein cavity visualized by x-ray crystallography. Proceedings of the National Academy of Sciences 96(1): 103–108. 10.1073/pnas.96.1.103 PMC15100 9874779

[20] Danley DE (2006) Crystallization to obtain protein-ligand complexes for structure-aided drug design. Acta Crystallographica D 62(6): 569–575. 10.1107/S0907444906012601 16699182

[21] McNae IW, Kan D, Kontopidis G, Patterson A, Taylor P, et al (2005) Studying protein–ligand interactions using protein crystallography. Crystallography Reviews 11(1): 61–71.

[22] Dahlquist FW, Raftery MA (1968) A nuclear magnetic resonance study of association equilibrium and enzyme-bound environments of N-acetyl-D-glucosamine anomers and lysozyme. Biochemistry 7(9): 3269–3277. 5684348 10.1021/bi00849a033

[23] Seemann KM, Kiefersauer R, Jacob U, Kuhn B (2012) Optical pH Detection within a Protein Crystal. The Journal of Physical Chemistry B 116(33): 9873–9881. 22834887 10.1021/jp2103512

[24] Hodgson KO (chairman), Anderson WF, Berman L, Fischetti R, Hendrickson WA, et al. (2009) Workshop of the National Institutes of Health National Center for Research Resources and the National Institute of General Medical Sciences on plans for support of future life science synchrotron research at NSLS-II, final report. June 4–5, 2009, Bethesda, MD.

